# Double-blind, randomized, multicentre, and active comparator controlled investigation of the effect of Pioglitazone, Metformin, and the combination of both on cardiovascular risk in patients with type 2 diabetes receiving stable basal insulin therapy: the PIOCOMB study

**DOI:** 10.1186/1475-2840-10-65

**Published:** 2011-07-14

**Authors:** Markolf Hanefeld, Andreas Pfützner, Thomas Forst, Iris Kleine, Winfried Fuchs

**Affiliations:** 1 GWT-TUD mbH, Fiedlerstr. 34, 01307 Dresden, Germany; 2 Ikfe GmbH, Parcusstr. 8, 55116 Mainz, Germany; 3 Takeda Pharma GmbH, Viktoriaallee 3 - 5, 52066 Aachen, Germany

## Abstract

**Background:**

We analyzed specific effects of an add-on therapy with pioglitazone compared to metformin and their combination in patients with basal insulin treatment on biomarkers of CV risk.

**Methods:**

In this double-blind, randomized, multicentre, active comparator controlled trial, 121 patients with type 2 diabetes were enrolled. Inclusions: treatment with basal insulin, HbA_1C _6.5% - 8.5%, age 30 - 75 years. After glargine therapy over 2 weeks for titration towards FBG ≤ 7.8 mmol/L, patients received either (A) bid 850 mg metformin (n = 42), (B) bid 15 mg pioglitazone (n = 40), or (C) 30 mg pioglitazone plus 1.7 g metformin (n = 39) over 6 months. Matrix Metal Proteinase 9 (MMP-9) was primary objective, together with biomarkers of CV risk.

**Results:**

Pioglitazone (B) reduced MMP-9 versus baseline by 54.1 + 187.1 ng/mL, with metformin (A) it was increased by 49.6 + 336.2 ng/mL (p = 0.0345; B vs. A), and with the combination of both (C) it was decreased by 67.8 + 231.4 ng/mL (A vs. C: p = 0.0416; B vs. C: p = 0.8695). After logarithmic transformation due to high variances the exploratory results showed significance for A vs. B (p = 0.0043) and for A vs. C (p = 0.0289).

Insulin dosage was reduced by 7.3 units in group B (p < 0.0001), by 6.0 units in C (p = 0.0004), but was increased by 2.5 units (p = 0.1539) in A at follow up. Reduction in hs-CRP was significant within treatment groups for B (p = 0.0098) and C (p < 0.0001), and between the groups for A vs. C (p = 0.0124). All three single regimens reduced PAI-1. Adiponectin was significantly elevated in B and C (p < 0.0001) and between-groups. HbA_1C _was only significantly decreased in the combination group. No significant effects were observed for NFkB and PGFα. peripheral edema were seen in 11.9% vs. 40.0% vs. 20.5%, and weight change was -0.7 kg vs. +4.3 kg vs. +2.7 kg (A vs. B vs. C).

**Conclusions:**

Addition of pioglitazone but not of metformin reduces MMP-9, hs-CRP and increased insulin sensitivity and adiponectin in this study. The combination of both had no additional effect on inflammation. Pioglitazone is suggested to be a rational add-on therapy to basal insulin in patients with high CV risk.

## Background

Along with control of hyperglycemia, treatment of patients with type 2 diabetes is aimed to reduce the elevated risk of cardiovascular disease (CVD) by correcting dyslipidemia, hypertension, low grade inflammation, and abnormalities in blood coagulation. International guidelines recommend the combination of metformin with basal insulin if monotherapy with metformin does not reach the target [1]. Combinations of insulin with pioglitazone and with metformin plus pioglitazone are considered as less validated therapies. So far efficacy and safety of pioglitazone in patients with stable treatment of basal insulin have not been assessed in comparative trials with metformin or metformin plus pioglitazone. Nevertheless, there are some reports on the effect of an add-on therapy with pioglitazone in patients with poorly controlled type 2 diabetes under intensified insulin therapy demonstrating that addition of pioglitazone significantly improved not only glycemic control but also had a positive effect on some major CV risk factors. Data from outcome studies suggest that metformin [2] and pioglitazone [3] can reduce CV events and progression of atherosclerosis. Insulin treatment, however, had no significant effect on cardiovascular outcome in the UKPDS [2].

Due to the absence of outcome data for a combination treatment of OAD with insulin, evaluation of CV risk markers can be used as surrogate parameters. So far a benefit/risk ratio for the comparator drugs as add-on therapy to insulin was only evaluated in patients with HbA_1C _> 8% [4]. With an HbA_1C _< 7% recommended by the ADA [5] and international guidelines, combination treatment under routine daily conditions is already considered for HbA_1C _between 7 and 8%. For this common clinical scenario determination of biomarkers of cardiovascular risk may help to evaluate possible benefits of combination treatment in high-risk patients. Besides hs-CRP [6,7], the matrix metalloproteinase 9 (MMP-9) is a known marker of inflammation produced by macrophages which contributes to vascular remodelling and transformation of stable to unstable plaques. Although MMP-9 seems to reflect an overall burden of vascular disease in type 2 diabetes patients [8-13] it is still controversially discussed to use it as a reliable surrogate marker of CV risk to date [14-16]. Hence, the primary outcome measure of the underlying double-blind, multi-centre, randomized, parallel three-arm trial was to investigate the effect of pioglitazone in comparison to metformin and the combination of both on MMP-9 together with a spectrum of established risk factors and biomarkers of inflammation during a 6-month therapy in type 2 diabetes patients pre-treated with insulin in order to use the generated data for evaluation of possible beneficial effects of comparator drugs as add-on treatment on cardiovascular risk. Clinical results and adverse events/side effects were secondary objectives.

## Methods

### Study Design and Patients

**Figure 1:**
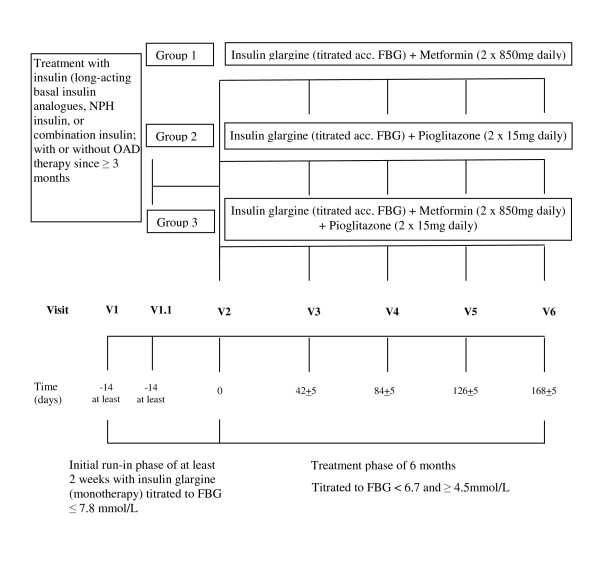
**Chart of Study Design**.

Type 2 diabetes patients treated with insulin (i.e., long acting basal insulin analogs, NPH insulin or combination insulin) in 1-2 daily doses for at least 3 months with or without oral antidiabetic drugs (OAD) except thiazolidinediones (TZD) prior to study entry were considered at an age between 30 and 75 years, with a BMI of ≥ 25 kg/m^2^, and if the baseline HbA_1C _was between 6.5 and 8.5%. Major exclusion criteria were uncontrolled hypertension, cardiovascular events within the previous year, and well established contraindications for metformin or pioglitazone regimens. An overview of the complete study design is presented in Figure 1. A list of participating sides is added in appendix (additional file 1)

The study was approved by the Saxonian Ethics committee. The Patients gave informed consent to take part in the study.

### Medical history and physical examination

Clinical evaluations and safety assessments including vital signs were done at screening, at randomisation, during study therapy, and at the last follow-up visit. Blood efficacy parameters were analysed in two central laboratories, blood safety parameters in local units. Body weight, insulin dosage, edemas, hypoglycemic episodes, and other possible side effects were recorded at each visit.

During an initial run-in phase of at least 2 weeks pre-existing insulin glargine therapy was titrated to an FBG of ≤ 7.8 mmol/L and other oral antidiabetics were stopped. If another insulin was used as previous therapy further treatment used insulin glargine. Patients were advised to measure FBG daily in order to adjust the insulin glargine dosage during the 6-month study treatment phase to a titration target of FBG between ≥ 4.5 and ≤ 6.7 mmol/L based on the investigator's discretion.

### Laboratory methods

Efficacy laboratory analyses were done according to each manufacturer's instructions in 2 central units and provided MMP-9 as primary parameter (ELISA of R&D systems, Wiesbaden/Germany). Secondary variables were: renal function (urinary albumin and creatinine excretion, crea./alb.-ratio, GFR), lipid profile (LDL/HDL/triglycerides: enzymatic/GPO-PAP, Selectra of Greiner Diagnostics, Bahlingen/Germany), metabolic markers (HbA_1C_, glucose, insulin, HOMA-S, and adiponectin which was measured by manual ELISA, Anthos, IBL, Hamburg/Germany), further inflammatory markers (hs-CRP: turbidimetry with Falcor 350 by Menarini, Neuss/Germany; PAI-1 with ELISA reader of American diagnostics, Pfungstadt/Germany), oxidative stress (urinary 8iso PGF_2α _by manual ELISA with Anthos of IBL, Hamburg/Germany), and insulin dosage. Patients with hs-CRP values >10 mg/dL were excluded from analysis due to possible distortion by general inflammations. Safety laboratory analyses yet included hematology, liver enzymes, potassium, creatinine, and CK.

### Statistical analysis

Efficacy analyses were done for the full analysis set (ITT), safety issues for the all patients treated (safety) analysis set. The primary statistical analysis was the testing of the effect of pioglitazone in comparison to metformin and the combination of both on a possible change of MMP-9 after a 6-month treatment compared to baseline. Confirmatory statistics was based on 2-sided t-tests for two independent samples calculated with the parameter estimates of an analysis of covariance (ANCOVA), using the primary parameter as dependent variable, the treatment group (with two levels) and centre as fixed factors, and the baseline MMP-9 value as covariate. Patients who terminated treatment before end of 6 months were considered with their individual last value under study medication (LOCF). Missing baseline MMP-9 values were not replaced.

The primary confirmatory analysis concerned the final statistical evaluation (second stage), was planned after completion of the 6-month visit, and was clearly to be distinguished from supporting secondary, exploratory evaluations. All p-values and confidence levels of additional inferential statistics were interpreted exploratorily. The confirmatory statistical evaluation was performed using a two-stage group-sequential Pocock design since the study design provided one interim and the final analysis. Two null-hypotheses were tested starting with H_11 _vs. H_12 _(C vs. A) at a significance level of α = 0.0307 (2-sided) for both study stages. A second test, H_21 _vs. H_22_, (B vs. A) was to be done confirmatorily only if the first test reached statistical significance. The third hypothesis (H_31 _vs. H_32_; C vs. B) was analyzed analogously in the exploratory sense only.

## Results

### Demographics and Baseline

A total of 121 patients on stable therapy with glargine insulin were randomized to receive additional treatment with metformin (850 mg bid; n = 42), pioglitazone (15 mg bid; n = 40), or the combination of both (n = 39). 39 patients in the metformin arm (A), 37 with pioglitazone (B), and 37 for the combination (C) were valid for intention to treat (i.e. full analysis set n = 113). Thus, 8/121 patients (6.6%; 3 vs. 3 vs. 2) were excluded as they failed to provide either evaluable baseline or at least one post-baseline value for MMP-9. 74/121 patients (61.1%) were male (23 vs. 25 vs. 26) and 47/121 patients (38.8%) were female (19 vs. 15 vs. 13), all Caucasians. Patient demographics (Table 1**) **showed a balance in major baseline characteristics at the time of randomization. The level of diabetes control with insulin glargine monotherapy during run-in resulted in HbA_1C _of 7.3% for all 3 groups. Patients had a diabetes history of >11 years and a 86% prevalence of hypertension. Average baseline results revealed FBG of 8.35 (± 2.17) mmol/L, hs-CRP of 3.21 (± 2.54) mg/L, and MMP-9 of 566.0 (± 266.2) ng/mL, representing a high-risk population for CVD despite suboptimal therapy with insulin.

**Table 1:** Demographic and Baseline Data

Baseline Parameter	Items	Total; n = 121	Metformin n = 42	Pioglitazone n = 40	MET + PIO n = 39
Sex	Male (%)	74 (61.2)	23 (54.8)	25 (62.5)	26 (66.7)
	Female (%)	47 (38.8)	19 (45.2)	15 (37.5)	13 (33.3)
Age [years]	Mean (SD)	63.0 (7.5)	64.2 (7.3)	61.5 (7.1)	63.3 (7.9)
Weight [kg]	Mean (SD)	92.5 (17.3)	89.4 (13.8)	91.9 (16.2)	96.6 (20.9)
BMI [kg/m^2^]	Mean (SD)	32.2 (5.3)	31.8 (5.0)	31.7 (4.3)	33.1 (6.4)
Duration of Diabetes Type 2 in [years]	Mean (SD)	11.1 (6.2)	12.3 (6.8)	9.8 (5.8)	11.0 (5.7)
	Median	10.0	11.0	8.0	10.0
	Range	1.0 - 31.5	3.0 - 31.5	1.0 - 28.0	1.3 - 29.0
SBP [mmHg]	Mean (SD)	137.5 (14.0)	138.8 (16.1)	137.0 (10.7)	136.5 (14.9)
DBP [mmHg]	Mean (SD)	78.1 (8.4)	78.5 (10.1)	77.5 (7.5)	78.2 (7.2)
FBG [mmol/L]	Mean (SD)	8.35 (2.17)	8.01 (1.96)	8.83 (2.52)	8.22 (1.95)
Insulin units	Mean (SD)	36.3 (20.9)	36.3 (20.1)	37.7 (22.9)	34.9 (19.9)
HbA_1C _[%]	Mean (SD)	7.34 (0.53)	7.35 (0.53)	7.33 (0.53)	7.34 (0.54)
MMP-9 [ng/mL]	Mean (SD)	566.0 (266.2)	589.7 (309.1)	530.9 (226.4)	576.5 (257.1)
hs-CRP [mg/L]	Mean (SD)	4.19 (4.14)	4.77 (4.91)	3.98 (3.43)	3.76 (3.93)
hs-CRP ≤ 10 [mg/L];	Mean (SD)	3.21 (2.54)	3.17 (2.37)	3.41 (2.87)	3.05 (2.39)
Creatinine [mg/dL]	Mean (SD)	0.84 (0.19)	0.81 (0.19)	0.86 (0.17)	0.86 (0.22)
Conc. BP-lower. ther.	n (%)	106 (87.6)	39 (92.9)	37 (92.5)	30 (76.9)
Conc. lipid-lower. ther.	n (%)	59 (48.8)	22 (52.4)	19 (47.5)	18 (46.2)
Conc. hypertension	n (%)	104 (86.0)	37 (88.1)	36 (90.0)	31 (79.5)
Conc. CHD	n (%)	23 (19.0)	13 (31.0)	7 (17.5)	3 (7.7)

Percentages are based on the total number of patients per group; SD: standard deviation; BMI: body mass index; SBP: systolic blood pressure; DBP: diastolic blood pressure; FBG: fasting blood glucose; HbA_1C_: haemoglobin A_1c_; MMP-9: matrix metallo proteinase-9; conc.: concomitant; lower.: lowering; ther.: therapy; CHD: coronary heart disease.

### Efficacy - Biomarkers of Cardiovascular Risk

For MMP-9 and most of the risk factors the additional use of pioglitazone was associated with more favourable effects compared to treatment with metformin (Table 2**)**. Pioglitazone (B) reduced MMP-9 by 54.1 + 187.1 ng/mL whereas metformin (A) increased MMP-9 by 49.6 + 336.2 ng/mL (p = 0.0345; B vs. A). The combination of both (C) was associated with a decrease in MMP-9 of 67.8 + 231.4 ng/mL (p = 0.0416, A vs. C and p = 0.8695, B vs. C). Logarithmic transformation for stabilization of the observed high variances revealed a rise of 0.1 + 0.5 ng/mL for A and a decrease of 0.1 + 0.5 ng/mL for both B and C. Now, exploratory p-values for the between-group comparison using the 3-group ANCOVA model reached significance for A vs. B (p = 0.0043) and for A vs. C (p = 0.0289), based on the pre-defined significance level of α = 0.0307.

**Table 2:** Course of Efficacy Parameters - Absolute Values

Secondary Efficacy Parameter [Unit]	absolute values during the study period: baseline vs. LOCF (full analysis set, n = 113); displayed as 'arithmetic means ± standard deviation (medians)*; n patients'*					
	
	A: Metformin (n = 39)		B: Pioglitazone (n = 37)		C: MET + PIO (n = 37)	
	
	Baseline^a^	LOCF	Baseline^a^	LOCF	Baseline^a^	LOCF
**Fasting glucose** **[mmol/L] (2)**	7.97 ± 1.98 (8.10); *39*	7.32 ± 1.90 (6.79); 39 *	8.73 ± 2.58 (8.40); *37*	7.34 ± 1.58 (6.99); 37 *	8.21 ± 1.96 (7.81); *37*	6.52 ± 1.48 (6.33); 37 *

**Fasting insulin** **[μIU/mL] (1) (2)**	10.44 ± 9.50 (7.20); *39*	12.43 ± 10.48 (9.20); 39 *	10.98 ± 7.71 (9.30); *37*	7.00 ± 4.41 (5.30); 37 *	8.49 ± 6.94 (5.90); *35*	6.10 ± 4.25 (4.60); 35 *

**HbA_1C _(2) (3)** **[%]**	7.33 ± 0.53 (7.30); *39*	7.23 ± 0.66 (7.10); *39*	7.35 ± 0.54 (7.20); *37*	7.19 ± 0.73 (7.20); *37*	7.34 ± 0.55 (7.30); *37*	6.85 ± 0.75 (6.70); 37 *

**HOMA-S (1) (2)** [mmol*mU/L^2^]	3.87 ± 3.89 (2.38); *39*	4.14 ± 3.84 (2.64); *39*	4.60 ± 3.93 (2.93); *37*	2.39 ± 1.79 (1.85); 37 *	3.40 ± 3.73 (1.97); *35*	1.80 ± 1.30 (1.22); 35 *

**MMP-9** **[ng/mL]**	601.7 ± 317.0 (544.8); *39*	651.3 ± 365.3 (543.3); *39*	535.0 ± 214.9 (533.7); *37*	480.9 ± 232.4 (443.2); *37*	581.8 ± 260.7 (504.5); *37*	514.0 ± 219.5 (474.3); *37*

**MMP-9 - Ln** **[ng/mL] (1) (2)**	6.3 ± 0.6 (6.3); *39*	6.4 ± 0.5 (6.3); *39*	6.2 ± 0.4 (6.3); *37*	6.1 ± 0.5 (6.1); 37 *	6.3 ± 0.4 (6.2); *37*	6.2 ± 0.4 6.2); *37*

**hs-CRP ≤ 10 (2)** **[mg/L]**	3.22 ± 2.43 (2.32); *33*	2.99 ± 2.42 (2.00); *33*	3.30 ± 2.73 (2.49); *35*	2.57 ± 2.07 (1.50); 35 *	2.62 ± 1.79 (1.99); *34*	1.78 ± 1.06 (1.46); 34 *

**Leucocytes** **[/nL] (1) (2)**	7.46 ± 1.86 (7.19); 39	7.26 ± 1.83 (6.90); 39	7.01 ± 1.65 (6.90); 37	6.01 ± 1.54 (5.90); 37 *	6.94 ± 1.81 (6.63); 37	6.10 ± 1.55 (6.18); 37 *

**NFkB** **[RLU]**	1.248 ± 0.756 (0.785); 38	1.228 ± 0.688 (0.805); 38	1.024 ± 0.630 (0.745); 36	0.992 ± 0.588 (0.705); 36	1.172 ± 0.707 (0.760); 35	1.154 ± 0.703 (0.750); 35

**PAI-1** **[ng/mL]**	71.2 ± 23.5 (70.0); *39*	61.2 ± 27.7 (54.4); 39 *	71.4 ± 25.7 (76.3); *37*	62.0 ± 29.9 (59.7); 37 *	70.9 ± 27.8 (76.0); *36*	53.3 ± 30.4 (54.5); 36 *

**Adiponectin** **[mg/L] (1) (2)**	4.43 ± 2.61 (4.16); *39*	4.33 ± 2.34 (4.00); *39*	4.29 ± 2.69 (3.85); *37*	13.20 ± 8.81 (11.49); 37 *	4.83 ± 3.08 (4.11); *37*	13.42 ± 7.69 (10.94); 37 *

**E-Selectin** **[ng/mL] (1) (2)**	47.1 ± 18.7 (45.3); 39	46.5 ± 19.9 (39.2); 39	48.2 ± 17.4 (43.4); 37	43.6 ± 16.2 (38.7); 38 *	45.7 ± 16.7 (43.8); 37	42.0 ± 16.1 (41.2); 37 *

**PGFα** **[ng/mmol]**	164 ± 89 (147); *38*	186 ± 99 (168); *38*	171 ± 131 (130); *36*	186 ± 114 (144); *36*	140 ± 46 (133); *37*	162 ± 94 (138); *37*

**Total cholesterol** **[mmol/L]**	5.02 ± 0.99 (4.90);39	4.82 ± 0.90 (4.90);39	4.80 ± 0.89 (4.56);37	4.89 ± 0.89 (4.86);37	4.71 ± 0.77 (4.68);37	4.87 ± 0.86 (4.59);37

**LDL-cholesterol** **[mmol/L]**	3.21 ± 0.78 (3.11); *38*	3.08 ± 0.78 (3.12); *38*	3.08 ± 0.72 (2.93); *37*	3.10 ± 0.74 (3.01); *37*	3.05 ± 0.62 (3.09); *37*	3.11 ± 0.68 (3.07); *37*

**HDL-cholesterol** **[mmol/L] (2) (3)**	1.24 ± 0.27 (1.30); *38*	1.29 ± 0.26 (1.28); *38*	1.19 ± 0.39 (1.16); *37*	1.29 ± 0.42 (1.17); 37 *	1.19 ± 0.29 (1.18); *37*	1.41 ± 0.36 (1.42); 37 *

**Triglycerides** **[mmol/L]**	2.03 ± 2.63 (1.54); *39*	1.76 ± 0.70 (1.66); *39*	1.76 ± 0.86 (1.50); *37*	1.70 ± 1.04 (1.35); *37*	1.72 ± 0.80 (1.71); *37*	1.54 ± 0.63 (1.52); 37 *

**Crea./Albu. Ratio** **[mmol/mg]**	1.14 ± 0.76 (1.18); *33*	1.72 ± 3.12 (0.97); *33*	3.47 ± 14.55 (0.88); *33*	1.19 ± 1.00 (0.93); 33 *	1.89 ± 3.14 (0.80); *35*	1.54 ± 1.61 (0.96); *35*

**GFR** **[ml/min]**	114.2 ± 34.2 (104.5); *39*	115.8 ± 38.9 (108.7); *39*	116.9 ± 33.7 (105.0); *37*	115.3 ± 36.6 (106.7); *37*	118.7 ± 47.3 (106.0); *37*	117.2 ± 47.9 (106.0); *37*

**Mean insulin dos.; [units] (1) (2)**	35.2 ± 17.1 (32.0); *38*	37.7 ± 19.6 (35.2); *38*	34.5 ± 16.9 (33.0); *35*	27.2 ± 14.6 (25.9); *35*	35.4 ± 20.3 (32.4); *37*	29.4 ± 20.9 (24.2); *37*

MET = Metformin; PIO = Pioglitazone; Ln = logarithmic transformation; LOCF = last observation carried forward

**^a)^**: baseline = values of V2.2 (randomization) or screening as applicable; dos. = dosage

* = p < 0.0307 for within group comparisons

**(1) **= p < 0.0307 for MET vs. PIO; **(2) **= p < 0.0307 for MET vs. MET+PIO; **(3) **= p < 0.0307 for PIO vs. MET+PIO.

Reduction of hs-CRP was significant within-group for B (p = 0.0098) and C (p < 0.0001), and between-group for A vs. C (p = 0.0124). All three add-on treatments reduced PAI-1 within-group but without any significance for the between-group comparisons. No effects were observed on the level of fibrinogen with any of the 3 regimens. The same applies for PGFα and NFkB activity. Adiponectin was significantly increased in group B and C (p < 0.0001) without an effect for metformin alone. This was confirmed between the groups with p < 0.0001 for A vs. B and A vs. C.

**Figure 2:**
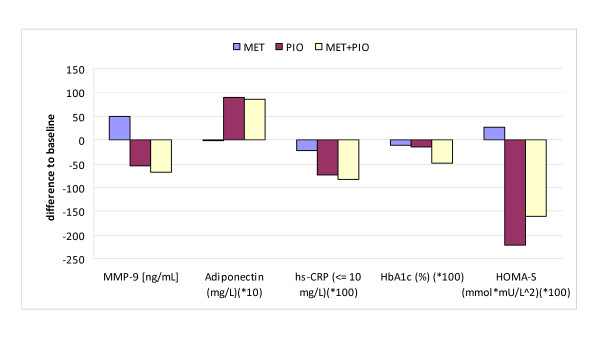
**MMP-9 and Secondary Efficacy: Mean Difference to Baseline after 6 months**.

Figure 2 illustrates the different effects of the three treatment regimens on MMP-9, insulin resistance and inflammation, indicating a clear superiority of pioglitazone with respect to biomarkers of inflammation, insulin resistance, and adiponectin.

### Efficacy - Metabolic and Hormonal Parameters

Metformin or pioglitazone had only minor effects on HbA_1C _(A: -0.11%, B: -0.15%) whereas the combination of both reached significance within-group (C: -0.49%, p < 0.0001), all versus baseline. The number of patients with a result for HbA_1C _of < 7% at randomization was 11/42 (26.2%; A) vs. 12/40 (30.0%; B) vs. 9/39 (23.1%; C). The pertinent result for the individual last visit was 17/42 (40.5%) vs. 13/40 (32.5%) vs. 25/39 (64.1%) of patients.

Fasting insulin and glucose were reduced in all 3 arms, whereas HOMA-S was improved only for B (p < 0.0001) and C (p < 0.0001). Thus, the between-group effect on HOMA-S was significant for A vs. B (p = 0.0001) and A vs. C (p < 0.0001). Eventually, mean insulin dosage was reduced by 7.3 units in group B (p < 0.0001) and by 6.0 units in C (p = 0.0004), but was increased in the metformin arm by 2.5 units (p = 0.1539). Thus, the between-group comparison on insulin dosage was significant for A vs. B (p < 0.0001) and for A vs. C (p = 0.0005). A minor but relevant increase in HDL-cholesterol was observed for both arms with pioglitazone (B: p = 0.0015; C: p < 0.0001). Between-group comparisons revealed a stronger increase in HDL for C vs. A (p = 0.0004) and C vs. B (p = 0.0162). A marked reduction of triglycerides was only observed for the combination arm (p = 0.0229). No such effects occurred with LDL-cholesterol. Excretion of 8isoPGF_2α_, an established indicator of oxidative stress, was not affected by addition of the study drugs. Moreover, only pioglitazone improved creatinin/albumine ratio in urine during the study period. However, GFR remained unchanged in all groups.

### Safety

Adverse events were documented in 99/121 patients (81.8%; 32 vs. 35 vs. 32, A vs. B vs. C) showing 383 (138 vs. 136 vs. 109) single events. Most frequently reported were nasopharyngitis in 32/121 (26.4%; 10 vs. 10 vs. 12), peripheral oedema in 29/121 (24.0%; 5 vs. 16 vs. 8), hypoglycemia in 28/121 (23.1%; 9 vs. 8 vs. 11), weight increase in 21/121 (17.4%; 3 vs. 11 vs. 7), and fatigue in 12/121 (9.9%; 5 vs. 1 vs. 6) patients. The calculated mean weight change was -0.7 kg in A, 4.3 kg in B, and 2.7 kg in C. Events assessed as related to study drug administration were mainly hypoglycemia in 26/121 (21.5%; 9 vs. 7 vs. 10), peripheral oedema in 26/121 (21.5%; 5 vs.14 vs. 7), weight increase in 21/121 (17.4%; 3 vs. 11 vs. 7), fatigue in 8/121 (6.7%; 4 vs. 1 vs. 3), and vertigo in 7/121 patients (5.8%; 4 vs.1 vs. 2). The better control of HbA_1C _in the combination group C was not associated with an increased risk of hypoglycemia. Premature termination due to an adverse event was seen in 9/121 patients (7.4%; 1 vs. 5 vs. 3) reporting 13 (4 vs. 5 vs. 4) events.

## Discussion

This prospective, randomized, controlled trial in patients with long-term type 2 diabetes on high cardiovascular risk under the conditions of stable insulin treatment reveals significant improvements with increased levels for biomarkers of lowgrade inflammation, insulin resistance, and associated CV risk factors, regarding the add-on therapy with pioglitazone and the combination of pioglitazone with metformin. As shown in previous studies [17-19] individual therapies have specific effects on non-traditional biomarkers of CV-risk, independent from good glycemic control.

We observed a clinically relevant reduction of MMP-9 activity only with pioglitazone and with the combination of pioglitazone plus metformin. Since change in FBG and HbA_1C _at follow-up was in the same range for add-on treatment with pioglitazone and metformin resp. these effects of the PPARγ agonist on MMP-9 may represent a direct effect rather than a consequence of minor improvement in glucose control. This finding is in accordance with previously published studies on combination therapy with insulin [17,18,20]. Furthermore, pioglitazone but not metformin reduced hs-CRP and increased adiponectin and the combination of both had no better effect on these emerging risk factors. Fibrinolysis activity measured with PAI-1, however, was improved for all three regimens without any differences between the groups. As expected, pioglitazone clearly reduced insulin resistance calculated by HOMA whereas metformin had no effect on insulin resistance in these patients treated with glargine insulin. Of notice, insulin dosage was reduced in both arms with pioglitazone by 7 and 6 units, respectively (p < 0.0001), whereas an enhancement of insulin consumption was observed for metformin to reach the target for FBG.

The results suggest that the observed effects of pioglitazone on inflammational biomarkers may be direct consequences of PPARγ activation. This hypothesis is supported by previously published results of a randomized controlled trial with non-diabetic patients suffering from cardiovascular diseases and elevated hs-CRP, where a significant beneficial effect of pioglitazone on hs-CRP, MMP-9 and PAI-1 could be demonstrated [21]. Oxydative stress is considered as major cause of diabetic complications [22]. Under the conditions of the present study with suboptimal diabetes control by stable insulin glargine treatment and the individual add-on therapy with OAD had no effect on 8iso PGF_2α _excretion. As shown by Monnier et al. [23] insulin treatment is able to normalize 8iso PGF_2α _excretion. The specific add-on therapy had only minor effects on lipids. Only HDL-C was significantly increased for the OAD combination vs. metformin alone and for within group comparisons. Accordingly, triglycerides were clearly lower at LOCF for the combination compared to metformin. Beneficial effects of add-on therapy with pioglitazone to intensive insulin therapy on dyslipidemia were reported for patients with HbA_1C _> 8% at baseline [24].

By extrapolation, our patients had a median diabetes duration of 11 years and a high rate of CV comorbidities treated with a polypharmacy. HbA_1C _at baseline was 7.34% with stable insulin glargine treatment but was associated with an elevated level of non-traditional CV risk factors such as MMP-9 and hs-CRP. Of notice, there is more and more evidence that MMP-9 serves as indicative for the presence of unstable plaques and activated macrophages [6,25,26]. Hence, our patients represent a high-risk group for CVD, despite suboptimum insulin treatment of type 2 diabetes and medical therapy of associated comorbidities.

As shown in the Jupiter study a reduction of hs-CRP by rosuvastatin resulted in a significant decrease of CV morbidity and mortality [27]. In the PERISCOPE study treatment with pioglitazone resulted in a non-progression of coronary plaques directly measured with quantitative angiography [3,28]. Thus, it is likely that by addition of the PPARγ agonist pioglitazone we may close a gap in protection of patients on high risk for CVD caused by increased inflammatory activity which is not controlled with insulin treatment alone.

Regarding the clinical results we are able to confirm a previously reported sparing effect on insulin dose which was not possible with metformin. A slightly lower baseline FPG in this group did not have a relevant clinical influence. With the triple treatment of insulin, pioglitazone, and metformin a significantly better control of HbA_1C _could be observed but no relevant differences were seen between the two dual combination groups with respect to CV risk factors. Better control of HbA_1C _< 7% with the combination was not associated with an increased rate of hypoglycemic events. In accordance to previous studies [29,30], a slight improvement of albuminuria was observed only for the add-on therapy with pioglitazone. No effect, however, was observed on GFR in all groups. In accordance to previously published studies with an add-on therapy of pioglitazone to insulin [3,4] we observed peripheral edemas in 40% of the patients treated with pioglitazone add-on therapy, as well as weight gain in 27.5%. However no serious adverse events were observed. Specifically no adverse effects on the left ventricular function occurred supporting the results of a recent tissue doppler imaging study investigating pioglitazone in type 2 diabetes patients with evidence of a left ventricular diastolic dysfunction [31].

## Conclusions

In patients with long term type 2 diabetes and suboptimal stable insulin treatment, the addition of pioglitazone but not metformin reduced the level of inflammatory biomarkers such as MMP-9 and hs-CRP and increased insulin sensitivity and adiponectin. The combination of pioglitazone with metformin resulted in better HbA_1C _and lipid control without added effect on inflammation, fibrinolysis, and renal function. No serious side effects were observed in any regimen but pioglitazone treatment was associated with more edema and weight gain as expected. Controlled clinical trials measuring cardiovascular endpoints are needed to compare risk benefit of individual add-on treatment with oral antidiabetic drugs to basal insulin, a question which is of high clinical relevance.

## List of abbreviations

ADA: American Diabetes Association; ANCOVA: Analysis of Covariance; BMI: Body-Mass Index; CHD: Coronary Heart Disease; CK: Creatine Kinase; CV(D): Cardiovascular (Disease); ELISA: Enzyme-linked Immuno-absorbent Assay; FBG: Fasting Blood Glucose; GFR: Glomerular Filtration Rate; GWT-TUD: Company for Science and Technology Transfer - Technical University Dresden GmbH; HbA_1C_: Haemoglobin A_1c_; HDL: High Density Lipoprotein; HOMA S: Homeostasis Model Assessment of insulin Sensitivity; hs-CRP: high-sensitivity C-Reactive Protein; ikfe: Institute for Clinical Research and Development GmbH; LDL: Low Density Lipoprotein; LOCF: Last Observation Carried Forward; MMP-9: Matrix Metallo Proteinase-9; NFkB: Nuclear Factor-kappa B; OAD: Oral Antidiabetic Drugs; PAI-1: Plasminogen Activator Inhibitor-1; PGFα: 8-iso Prostaglandine F2 alpha; PPAR: Peroxisome Proliferation Activating Receptor; TZD: Thiazolidinedione.

## Competing interests

**Markolf Hanefeld: **Honoraria speaker from TAKEDA, GSK, Sanofi-Aventis; Advisory Board: Sanofi-Aventis, GSK.

**Winfried Fuchs **is employee of TAKEDA Pharma Germany.

**Iris Kleine **is employee of TAKEDA Pharma Germany.

**Thomas Forst **received speaker funds and research supports from TAKEDA Pharma GmbH Aachen/Germany.

**Andreas Pfützner **received speaker funds and research supports from TAKEDA Pharma GmbH Aachen/Germany.

## Authors' contributions

Preparation, drafting, finalization, and submission of the present manuscript was done by the GWT-TUD mbH, Dresden/Germany: MH. The trial was sponsored and conducted by Takeda Pharma GmbH, Aachen/Germany: IK and WN. The ikfe GmbH, Mainz/Germany was responsible for the trial design as well as for development and preparation of the study protocol and the case report forms: AP and TF. We confirm that all authors read and approved the final manuscript.

## Supplementary Material

Additional file 1 **List of participating investigators and active study sites (n = 13)**. Contains Responsible Investigators and active German Study Centres (N = 13) and Randomized and Treated Patients (n = 121; final analysis/safety set). Click here for file
