# Supplementary material for: Double-blind, randomized, multicentre, and active comparator controlled investigation of the effect of Pioglitazone, Metformin, and the combination of both on cardiovascular risk in patients with type 2 diabetes receiving stable basal insulin therapy: the PIOCOMB study
Source: Cardiovasc Diabetol. 2011 Jul 14;10:65. doi: 10.1186/1475-2840-10-65 (PMC3160877; doi:10.1186/1475-2840-10-65)
Supplement: Additional file 1 — List of participating investigators and active study sites (n = 13). Contains Responsible Investigators and active German Study Centres (N = 13) and Randomized and Treated Patients (n = 121; final analysis/safety set). [file 1475-2840-10-65-S1.DOC]

List of participating investigators and active study sites (n=13)

| **Centre-Number** | **Responsible Investigators and active German Study Centres (N=13)** | **Randomized and Treated Patients (n=121; final analysis/safety set)** |
| --- | --- | --- |
| **01** | Prof. M. Hanefeld, MD/PhD; KKS GWT-TUD  01307 Dresden | 21 patients; Random-Nos.: 1-6, 133-138, 151-156, 181, 182, 184 |
| **02** | Prof. A. Pfützner MD/PhD; Ikfe GmbH  55166 Mainz | 56 patients; Random-Nos.: 7-12, 19-48, 67-78, 139-146 |
| **03** | Prof. D. Tschöpe, MD/PhD  32545 Bad Oeynhausen | 1 patient;  Random-No.: 13 |
| **08** | A. Boustani, MD/PhD  65582 Diez | 8 patients;  Random-Nos.: 103-108, 157, 158 |
| **09** | H. Braun, MD/PhD  10409 Berlin | 8 patients;  Random-Nos. 55-60, 193, 194 |
| **10** | Claudia Büttner, MD/PhD  10117 Berlin | 5 patients;  Random-Nos.: 61-65 |
| **12** | S. Kress, MD/PhD  76829 Landau | 6 patients;  Random-Nos.: 91-96 |
| **14** | Ilka Simon-Wagner, MD/PhD  96215 Lichtenfels | 1 patient;  Random-No.: 127 |
| **15** | Huptas, MD/PhD  45219 Essen | 1 patient;  Random-No.: 85 |
| **18** | Klara Wilhelm, MD/PhD  04103 Leipzig | 3 patients;  Random-No.: 109, 110, 111 |
| **19** | A. Barakat, Dipl. MD  47051 Duisburg | 5 patients;  Random-Nos.: 187-191 |
| **21** | M. Esser, MD/PhD  45219 Essen | 2 patients;  Random-Nos.: 169, 170 |
| **22** | H.-J. Rüßmann, MD/PhD  46537 Dinslaken | 4 patients;  Random-Nos.: 199-202 |
